# The Beginning of Ending Hepatitis C Virus: A Summary of the 26th International Symposium on Hepatitis C Virus and Related Viruses

**DOI:** 10.3390/v12030302

**Published:** 2020-03-11

**Authors:** Eui-Cheol Shin, Ji Won Han, Wonseok Kang, Takanobu Kato, Seong-Jun Kim, Jin Zhong, Seungtaek Kim, Su-Hyung Park, Pil Soo Sung, Koichi Watashi, Jun Yong Park, Marc P. Windisch, Jong-Won Oh, Takaji Wakita, Kwang-Hyub Han, Sung Key Jang

**Affiliations:** 1 Graduate School of Medical Science and Engineering, Korea Advanced Institute of Science and Technology (KAIST), Daejeon 34141, Korea; tmznjf@kaist.ac.kr (J.W.H.); park3@kaist.ac.kr (S.-H.P.); 2 Department of Medicine, Samsung Medical Center, Sungkyunkwan University School of Medicine, Seoul 06351, Korea; wskang.md@gmail.com; 3 Department of Virology II, National Institute of Infectious Diseases, Tokyo 162-8640, Japan; takato@nih.go.jp (T.K.); kwatashi@niid.go.jp (K.W.); wakita@nih.go.jp (T.W.); 4 Center for Convergent Research of Emerging Virus Infection, Korea Research Institute of Chemical Technology, Daejeon 34114, Korea; juni0080@gmail.com; 5 Unit of Viral Hepatitis, Institut Pasteur of Shanghai, Chinese Academy of Sciences, Shanghai 200031, China; jzhong@ips.ac.cn; 6 University of Chinese Academy of Sciences, Beijing 100049, China; 7 Zoonotic Virus Laboratory, Institut Pasteur Korea, Seongnam 13488, Korea; seungtaek.kim@ip-korea.org; 8 Division of Gastroenterology and Hepatology, Department of Internal Medicine, College of Medicine, Seoul St. Mary’s Hospital, The Catholic University of Korea, Seoul 06591, Korea; pssung@catholic.ac.kr; 9 Department of Internal Medicine, Yonsei University College of Medicine, Seoul 03722, Korea; drpjy@yuhs.ac (J.Y.P.); gihankhys@yuhs.ac (K.-H.H.); 10 Applied Molecular Virology Laboratory, Institut Pasteur Korea, Seongnam 13488, Korea; marc.windisch@ip-korea.org; 11 Department of Biotechnology, Yonsei University, Seoul 03722, Korea; jwoh@yonsei.ac.kr; 12 Department of Life Sciences, Pohang University of Science and Technology, Pohang 37673, Korea; sungkey@postech.ac.kr

**Keywords:** hepatitis C virus, antivirals, vaccine, flavivirus, liver diseases

## Abstract

Hepatitis C virus (HCV) infects ~71 million people worldwide, and 399,000 people die annually due to HCV-related liver cirrhosis and hepatocellular carcinoma. The use of direct-acting antivirals results in a sustained virologic response in >95% of patients with chronic HCV infection. However, several issues remain to be solved to eradicate HCV. At the 26th International Symposium on Hepatitis C Virus and Related Viruses (HCV2019) held in Seoul, South Korea, October 5–8, 2019, virologists, immunologists, and clinical scientists discussed these remaining issues and how we can achieve the elimination of HCV.

## 1. Introduction

Approximately 71 million people worldwide are chronically infected with hepatitis C virus (HCV), and infected individuals are at an increased risk of liver cirrhosis and hepatocellular carcinoma (HCC) [1]. Approximately 399,000 people are estimated to die annually from HCV-related liver cirrhosis and HCC [2]. Currently, treatment with direct-acting antivirals (DAAs) achieves a sustained virologic response (SVR) in more than 95% of patients infected with HCV [1]. Highly efficacious antivirals led the World Health Organization (WHO) to strive to eliminate HCV by 2030, though no effective vaccine against HCV is currently available.

The 26th International Symposium on Hepatitis C Virus and Related Viruses (HCV2019) was held in Seoul, South Korea, October 5–8, 2019. At this symposium, virologists, immunologists, and clinical scientists discussed remaining issues in the field and how we can achieve the elimination of HCV. The key contents are summarized as follows:

1. Immunology and vaccinology
  - Incomplete recovery of memory T cells after SVR, which allows re-infection of HCV in successfully treated subjects.
  - Significance of both broadly neutralizing antibodies and T cells in HCV-specific immune responses, which is important for prophylactic HCV vaccine development.
  - Suggestion for controlled human infection models in HCV vaccine trials.
2. Virology and virus-host interaction
  - Persistence of epigenetic changes in the liver, even after SVR, which may lead to the development of HCC.
  - Altered lipid metabolism during the HCV life cycle in infected hepatocytes.
3. Clinical issues and public health
  - Issues in difficult-to-cure patients/viruses (genotype 3 and unusual subtypes).
  - Enhanced HCV testing and linkage to care.
  - Importance of rapid diagnostic tests.

Importantly, the results of the first prophylactic HCV vaccine efficacy trial (NCT01436357) was presented at HCV2019. The trial failed in terms of efficacy but was successful in terms of lessons for prophylactic HCV vaccine development.

## 2. Hepatocellular Carcinoma, Cirrhosis, and Pathogenesis

In her plenary lecture, Young Nyun Park summarized the accumulation of pathological and molecular changes during hepatocarcinogenesis, from cirrhosis to low-grade dysplastic nodule, high-grade dysplastic nodule, and early HCC, and the final progression to HCC. In his keynote lecture, Ju-Seog Lee provided a comprehensive overview of the genomics of HCC, highlighting the distinctive genomic subtype associated with HCV infection which may dictate clinical outcomes and treatment response. Several groups focused on HCV-induced alterations in epigenetic and gene expression signatures on the host cell which are implicated in liver pathogenesis. Griffin et al. showed that hepatic differentiation of patient-derived liver progenitor cells is diminished by HCV infection ex vivo, which was related to HCV-induced perturbation of Hippo signaling, which is frequently dysregulated in hepatocarcinogenesis. The majority of differentially expressed genes, as assessed by transcriptomic analysis, were not altered after eradication of the virus, suggesting a potential link between HCV-imprinted gene expression and epigenetic changes underlying HCV-related hepatocarcinogenesis. Gal-Tanamy et al. performed integrated transcriptome, epigenome, and kinome screens and determined that activation of epidermal growth factor receptor (EGFR) following HCV infection upregulates the expression of invasion-related genes [3]. Regarding their results, HCV NS3 protease cleaves and inactivates T-cell protein tyrosine phosphatase (TCPTP), a phosphatase that inhibits EGFR signaling, contributing to HCV-induced invasion phenotypes. Another area of interest was the interplay between HCV and host cell metabolism. Based on previous findings that HCV infection silences protein tyrosine phosphatase receptor delta (PTPRD) expression [4], Lupberger et al. analyzed the transcriptome data from *Ptprd* knock-out mice and human liver. They showed that PTPRD silencing by HCV might contribute to the development of HCV-associated metabolic disease, which is linked to deregulated STAT3 signaling and impaired peroxisomal function via suppression of peroxisome proliferator-activated receptor alpha (PPARA). Using in vitro models, Ohta et al. furthered our understanding of intracellular iron overload in HCV infection by showing that CREBH (cyclic adenosine monophosphate-responsive element-binding protein H) activation triggered by HCV infection induces hepcidin expression through not only its recruitment to hepcidin promoter, but also through upregulation of the BMP (bone morphogenic protein)/SMAD pathway. On the other hand, Matsui et al. were interested in the mechanism underlying the formation of large lipid droplets in HCV infection and reported that HCV NS3/4A specifically cleaves spartin/SPG20 protein, which in turn inhibits the ubiquitination of adipophilin by atrophin-1-interacting protein 4 (AIP4) E3 ligase and decreases lipid droplet turnover, suggesting a mechanism for NS3/4A-induced steatosis. Liu et al. infected common marmosets with HCV/GBV-B chimeras containing HCV envelope and/or core proteins to identify the potential role of the HCV core in hepatic inflammation. Using transcriptomic analysis and in vitro functional assays, they demonstrated that the HCV core induces interleukin (IL)-32 expression in hepatic cells via the PI3K pathway and may play a major role in the development of HCV-related severe hepatitis. Finally, in a special lecture, Seung Kew Yoon presented a detailed overview of recent progress in the treatment of HCC and the remaining challenges and knowledge gaps. Thus, this session expanded our understanding of the complex genomic and molecular networks that contribute to HCV-related liver disease pathogenesis. 

## 3. Viral Entry and Replication

Ralf Bartenschlager delivered the plenary lecture highlighting that HCV is potentially one of the best tools to study cell biology. He elaborated on crucial host factors associated with HCV replication and assembly, and also mentioned the HCV-induced double-membrane vesicles (DMVs) that are essential for HCV assembly. Prentoe et al. presented the ’open’ and ’closed’ conformation of the HCV envelope, and mentioned that these structures are associated with the neutralization sensitivity or receptor dependency of HCV infection [5]. Zhang et al. described the anti-HCV effects of the interferon-stimulated gene *shiftless* (*SFL*). The mouse and guinea pig orthologs of SFL inhibit HCV, as does human SFL. Kalemera et al. reported that the entry-optimized variants of HCV with amino acid substitutions are less dependent on scavenger receptor class B type-I (SR-B1) at the entry step and more sensitive to neutralizing antibodies [6]. Abe et al. presented that HCV NS5A-ISGylation promotes HCV replication via an association with cyclophilin A. In the keynote lecture, Thomas Baumert reviewed recent progress on the association of key molecules with the HCV entry step and discussed the pathogenesis of this virus, highlighting the claudin-1-associated activation of EGFR signaling and the development of HCC. Ono et al. presented that microRNAs other than miR-122 also support HCV replication via formation and stabilization of the structure of the HCV internal ribosome entry site (IRES). Schenk et al. discussed the host factors that support the replication of a non-adaptive HCV strain in cell culture. They assessed the effects of the overexpression of SEC14L2 [7] and the inhibition of phosphatidylinositol-4-phosphate kinase III alpha and casein kinase I alpha (PI4KA/CKIα) on HCV replication in cell culture, finding that the functions of these factors overlap and are not additive. Tsukiyama-Kohara et al. reported that ribonucleotide reductase M2 stabilizes the NS5A protein by avoiding human homolog 1 of protein-linking integrin-associated protein and cytoskeleton (hPLC1)-dependent ubiquitination, supporting HCV replication [8]. In addition, Liang et al. reported that E3 ubiquitin ligase TRIM26 affects HCV genome replication via specific interactions with HCV NS5B RNA-dependent RNA polymerase. Frakolaki et al. described the enhanced replication of Flaviviridae viruses and reduced effect of antivirals in cell culture models under hypoxia. Overall, the findings presented in this session mainly focused on the host factors associated with HCV entry and replication steps and provided new information to aid understanding HCV infection and pathogenesis.

## 4. Viral Assembly and Virus–Host Interaction 

Lassen et al. investigated the effects of perilipin-2 (PLIN2) on HCV infection and lipid droplet (LD) morphogenesis in hepatocytes [9]. They showed that PLIN2 expression is required for trafficking core and NS5A proteins to LDs and the formation of functional low-density HCV particles prior to ApoE incorporation. Fukuhara et al. showed that NS1 protein plays an important role in flavivirus particle formation and the function of apolipoprotein and Erns in HCV and Pestivirus particle formation using ZIKV cDNA clone (ZIKV-HiBiT). Lee et al. showed that miRNA-99a, which regulates the mTOR pathway and cell survival, modulates HCV replication by blocking mTORC1-mediated de novo lipogenesis. Their data suggest that miRNA-99a ameliorates intracellular lipid accumulation by regulating the expression of SREBP-1c during HCV infection. Aiming to clarify the physiological relevance of activation of E3 ubiquitin ligase Itch in the HCV life cycle, Deng et al. presented that Itch silencing reduces extracellular HCV infectivity, but not intracellular infectivity. They showed that Itch is involved in the release of infectious HCV particles but not HCV RNA replication via VPS4A polyubiquitination. Analysis of the transcriptome and translatome by Gerresheim et al. using ribosome profiling (Riboseq) indicated that HCV infection induces a significant increase in the expression of roughly 30 host genes related to ER stress and HCV replication in HCV-infected Huh7.5 cells [10]. Nishioka et al. screened a flavone derivative library to select a specific inhibitor modulating aryl hydrocarbon receptor (AhR) and its downstream gene, cytochrome P450 family 1A1 (CYP1A1), and showed that two flavones reduce HCV assembly by disrupting LDs and repressing the transcriptional activity of AhR and CYP1A1 mRNA in HCV-infected Huh7 cells. Eu et al. investigated the role of a direct interaction between cholesterol and HCV viral proteins in the HCV life cycle, and found that efficient interactions between HCV NS2 protein and cholesterol results in altered subcellular localization in HCV-infected cells, arguing that specific binding of NS2 to cholesterol alters NS2 trafficking to sites of HCV assembly. Cao et al. reported that 100–200 circRNAs discovered from screening circRNA libraries were up- or down-regulated during HCV infection. They also showed that some circRNAs were produced from the viral genome, suggesting that they have pro- or anti-viral functions. Nguyen et al. sought to investigate the role of protein kinase C and casein kinase substrate in neurons protein 2 (PACSIN2) interactions with HCV NS5A protein. Their data indicate that HCV exploits the pro-viral cellular function of PACSIN2 to promote viral assembly [11]. Takeo et al. examined the potency of HCV NS3-specific nanobodies in HCV-infected cells and found that HCV NS3-specific nanobodies do not affect HCV RNA replication, but potently inhibit virus assembly at the stage of nucleocapsid envelopment. Currently, they are characterizing the NS3-binding properties of NS3 nanobodies and their effects on helicase RNA binding, unwinding, and ATPase activities.

## 5. Innate and Adaptive Immune Responses

This session focused mainly on the HCV-specific immunological functions of T cells and B cells/antibodies in the setting of repeating HCV infection or spontaneous/therapy-mediated viral clearance. In her keynote lecture, Naglaa Shoukry summarized the roles of B cells/antibodies and T cells in immune control of HCV infection, presenting their recent work on analyzing critical correlates of protective immunity during repeat HCV infection in people who inject drugs (PWIDs). Mazouz et al. reported transcriptomic analyses of PBMCs from patients who successfully resolved the primary HCV infection and did or did not clear subsequent infections. Damasio et al. analyzed T-cell responses in acute HCV infection in HIV^+^ patients treated with sofosbuvir/ribavirin. They found that, though HCV-specific CD4^+^, T-cell responses were often undetected in natural acute persisting HCV infection, they were always detectable in DAA-treated patients, regardless of the therapeutic outcome, suggesting that early DAA therapy protects HCV-specific CD4^+^ T cells from deletion in acute HCV infection. Han et al. analyzed the ex vivo functions of HCV-specific CD8^+^ T cells in chronic HCV patients treated with DAAs. They found that CD8^+^ T-cell responses were only transiently restored 4 weeks post-treatment and subsequently diminished, suggesting that DAA-mediated viral clearance is not able to fully restore CD8^+^ T-cell functions [12]. Hensel et al. performed single-cell sequencing on memory-like HCV-specific CD8^+^ T cells (CD127^+^PD1^+^) in chronic HCV patients and found that these memory-like HCV-specific CD8^+^ T cells possessed an exhausted signature and phenotype, which also suggests the incomplete restoration of CD8^+^ T cells in chronic HCV infection, even after viral clearance. Underwood et al. analyzed the B cells and antibody responses in 15 subjects with multiple repeat HCV infections. They found that a higher ratio of IgD^+^/IgG^+^ E2-positive B cells and less activated transcriptome profile correlates with protection from chronic progression of HCV re-infection, whereas the breadth of the HCV-specific antibody responses from earlier infection does not. Nishio et al. analyzed the antibodies and B cells in chronic HCV patients who had successfully cleared the infection using interferon- or DAA-based therapies. They found that all patients possessed functional HCV-specific B cells and broadly neutralizing antibodies, but the neutralizing ability decreased significantly after HCV clearance, regardless of the type of antiviral treatment. Chumbe et al. analyzed the sensitivity of a panel of 20 different HCVpp from genotype 1–6 to eight previously established broadly neutralizing antibodies; the neutralizing sensitivity of a given HCVpp appeared to be strain-specific but not genotype-specific. Augestad et al. analyzed the contribution of AS412, a linear epitope located immediately downstream of hypervariable region 1 (HVR1) of HCV E2 protein, to the neutralizing sensitivity. They found that AS412 is structurally flexible, and its β-hairpin conformation was associated with the previously defined “closed” and difficult-to-neutralize envelope conformation [5].

## 6. Emerging and Related Viruses

In the Emerging and Related Viruses session, research updates were presented for other hepatitis viruses, pestivirus (BVDV), and flaviviruses (Zika and West Nile viruses). The first keynote lecture given by Alexander Ploss focused on the progress in his hepatitis B virus (HBV) and hepatitis E virus (HEV) research. The hallmark of HBV infection is the formation of covalently closed circular DNA (cccDNA) in the nucleus of hepatocytes, which is the transcription template for HBV replication. He presented remarkable experimental results for the conversion from relaxed circular DNA (rcDNA), a mature viral genomic DNA, to cccDNA using yeast nuclear extracts. Furthermore, five host factors from yeast were shown to be sufficient for this rcDNA to cccDNA conversion. Mechanistically, this conversion process was similar to the lagging strand synthesis of double-stranded DNA. In his HEV presentation, he talked about another potential antiviral compound that has better antiviral efficacy than ribavirin. Brown et al. talked about the role of Zika virus (ZIKV) “M” protein as a viroporin. They demonstrated the formation of hexameric M complexes in in vitro experiments and molecular dynamics simulations in which its opening was modulated by a change in pH. M protein channels could be novel targets for antiviral development. Ramage et al. presented a mass spectrometry approach to identify protein–protein interactions and post-translational modification of proteins upon West Nile virus infection. The targets identified by mass spectrometry included interferon-stimulated genes and numerous other proteins with potential antiviral activity. They are exploring the roles of these proteins in flavivirus infection. Kokkonos et al. and Kui Li et al. talked about interactions between BVDV or ZIKV RNAs and miRNAs or E3 ligase TRIM56, respectively. These interactions are important in BVDV viral replication and host restriction in ZIKV infection.

## 7. Vaccines and New Antiviral Agents

In her keynote lecture, Andrea Cox presented the results from the first prophylactic HCV vaccine efficacy trial, a randomized, multicenter, double-blind, placebo-controlled study. The vaccine regimen is based on viral vectors consisting of a recombinant chimpanzee adenovirus 3 vector vaccine prime, followed by a recombinant modified vaccinia Ankara virus boost. Cox and colleagues found that this prime-boosted HCV vaccine regimen does not provide protection against chronic infection. However, compared to placebo, the vaccine regimen blunted the peak HCV RNA level in vaccine recipients. In terms of immunogenicity, 78% of the recipients induced an immune response, which is a less robust response than observed in an earlier study with healthy volunteers. Major et al. presented the immunogenicity of in vitro transcripts of the Venezuelan equine encephalitis virus (VEEV) self-amplifying RNA, which encodes the HCV NS3/4A protein. The data suggest that this self-amplifying RNA induces HCV-specific T-cell responses comparable to those induced by immunization with Ad5/NS34A. Prentoe et al. generated three different versions of soluble E1/E2 antigens in which with the transmembrane regions are deleted (i.e., fused peptide backbones), termed sE1E2 (E1 followed by E2), sE2E1 (E2 followed by E1), and sE21E (E2 followed by an inverted E1). They presented data suggesting that sE21E may represent an innovative HCV vaccine antigen approach that limits the induction of autologous, non-cross-genotype-reactive neutralizing antibody responses. Watanabe et al. reported a novel E2 monoclonal antibody that can inhibit HCV infection at a comparable level as already known neutralizing antibodies, HCV1, and AR3A, by HCVcc infection assay. Ohashi et al. reported on CH223191, a benzamide derivative that potently inhibits AhR transcriptional activity and prevents viral assembly by decreasing the amount of cellular LDs. Additionally, Andrea Cox highlighted that many countries are not on track to eliminate HCV by 2030, and she stressed the need to quickly cure chronically infected HBV and HCV patients; otherwise, the mortality rates will be higher than HIV, tuberculosis, malaria combined by 2040.

## 8. Enteric Hepatitis Viruses

The hepatitis A virus (HAV) and HEV pose significant global health threats, though they usually cause self-limited infections. Commercially available vaccines prevent infection with both viruses through the induction of antibodies that provide protective immunity [13]. In his keynote lecture, Christopher Walker mentioned two recent reports on immune-mediated liver injury in acute HAV infection. One report identified a deletion in *IL18BP* in a severely affected, HAV-infected child that results in excessive natural killer cell-mediated killing of hepatocytes [14]. The other report conveyed that bystander memory CD8^+^ T cells are activated by IL-15 without T-cell receptor engagement during acute HAV infection and exerts NKG2D-dependent innate-like cytotoxic activity [15]. In relation to the latter report, Seo et al. presented that IL-15 upregulates the expression of CCR5 in memory CD8^+^ T cells and contributes to their migration to the inflamed liver. Rha et al. presented that IL-15 also activates liver sinusoidal mucosal-associated invariant T (MAIT) cells to exert innate-like cytotoxicity. Regarding hepatocyte sensing of HAV after viral entry, Colasanti et al. confirmed that the HAV protease 3CD can only partially cleave TIR-domain-containing adapter-inducing interferon-β (TRIF) and cannot completely block the Toll-like receptor 3 (TLR3) response. On the other hand, HCV protease cannot cleave TRIF at all, and HCV infection robustly activates the TLR3 response. Regarding HEV research, Todt et al. presented the optimized protocol for the high titer production of enveloped and naked HEV particles. They introduced a single nucleotide variant in the polymerase domain of HEV and increased titers to a previously unreported 10^6^ focus forming units/mL. Using an ORF1-based transcomplementation system, Ju et al. identified two conserved regions within ORF1 and ORF2 that are critical for viral replication. Regarding the immunopathogenesis of HEV infection, Christopher Walker developed a macaque model of HEV infection and demonstrated that CD4^+^ T cells prevent persistent HEV infection. Two different infection outcomes after CD4^+^ T-cell depletion in HEV-infected macaques, persistent resolving and persistent non-resolving infection, were both associated with CD8^+^ T-cell exhaustion. Further research using the macaque HEV model is expected to reveal the mechanism of HEV persistence and HEV-mediated liver injury.

## 9. Issues in DAA Treatment

Jean-Michel Pawlotsky’s keynote lecture provided an overview of the current status of DAA therapy. In particular, he focused on difficult-to-cure patients due to virological reasons. Genotype 4r, which is a rare, African genotype, exhibited surprisingly low SVR (56%) with sofosbuvir/ledipasvir treatment. Another African genotype 1, had an SVR of 75%, and Chinese genotype 3b with liver cirrhosis achieved an SVR of 50% with sofosbuvir/velpatasvir. DAA re-treatment after failure of daclatasvir/asunaprevir treatment is another difficult-to-cure case. He stressed that one more class of antiviral agents is demanded to cover the remaining difficult-to-cure patients. Khera et al. identified soluble inflammatory mediators upregulated in acute hepatitis C patients depending on viral load, including IL-6 and TRAIL. These mediators were decreased by DAA treatment but were not normalized until 24 weeks of follow-up. Kim et al. found that the frequency of TNF-producing regulatory T (Treg) cells, which was higher in chronic hepatitis C patients, was transiently decreased following DAA treatment until week 8, but increased after that and returned to the original level 12 weeks after DAA treatment. Thus, inflammatory alterations in cytokines and Treg cells are sustained even after DAA treatment. Haba et al. employed an in vitro technology to detect giant unilamellar vesicles (GUVs) and addressed the role of NS5A on the formation of double-membrane vesicles (DMVs). An NS5A mutant consisting of the N-terminal amphipathic helix (AH) and domain I bound to GUVs and induced morphological remodeling. Daclatasvir inhibited GUV remodeling, NS5A-membrane interaction, and NS5A oligomerization, suggesting a direct suppression effect of NS5A inhibitors on DMV formation. Ma et al. presented the anti-HCV, pharmacokinetic, and toxicity profiles of fluoxazolevir, a new fusion inhibitor they are developing. They suggested that this compound inhibits the fusion process by targeting HCV E1 protein. Though fluoxazolevir or daclatasvir alone reduced the viral load approximately 2-log in an HCV-infected humanized chimeric mouse model, a combination of these agents resulted in a 6-log reduction. Notably, drug-resistant HCV in mice that do not respond to glecaprevir/pibrentasvir was decreased to undetectable levels 2 weeks after co-treatment with fluoxazolevir and glecaprevir/pibrentasvir. Fluoxazolevir can be a promising drug candidate to treat drug-resistant HCV, and would be the first DAA targeting the structural proteins of the virus. Dahari et al. proposed shortening the DAA treatment duration by applying their mathematical model to the analysis of viral kinetics [16,17,18]. Using the profile of the biphasic viral decline during DAA treatment in each patient, they calculated in real-time the duration until the viral load decreases below the cure boundary, showing for the first time that this personalized duration treatment approach is possible with DAAs in chronic hepatitis C patients.

## 10. HCV: 30 Years and Future

In his keynote lecture, T. Jake Liang summarized the progress in the 30 years since the discovery of HCV and the research directions for future HCV elimination. Although significant achievements have occurred in HCV research, many challenges remain, including difficult-to-treat patient populations, such as decompensated cirrhosis and genotype 3 infection, management of the major source of HCV transmission, including patients who inject drugs, and the residual risk for HCC after SVR. Since the discovery of HCV, the world has reached a public health landmark. The WHO set an ambitious goal of eliminating viral hepatitis by 2030. Finally, the current major challenge for the HCV field is the development of an effective vaccine, which is necessary for global control and elimination of HCV. 
